# Does Birth-Related Trauma Last? Prevalence and Risk Factors for Posttraumatic Stress in Mothers and Fathers of VLBW Preterm and Term Born Children 5 Years After Birth

**DOI:** 10.3389/fpsyt.2020.575429

**Published:** 2020-12-15

**Authors:** Dana Barthel, Ariane Göbel, Claus Barkmann, Nadine Helle, Carola Bindt

**Affiliations:** Department of Child and Adolescent Psychiatry, Psychotherapy, and Psychosomatics, University Medical Center, Hamburg-Eppendorf, Hamburg, Germany

**Keywords:** posttraumatic stress, preterm birth, risk factors, maternal/paternal, VLBW (very low birth weight)

## Abstract

Previous research suggests that the birth of a preterm child with very low birth weight (VLBW; <1,500 g) can be traumatic for both parents and lead to short-term consequences like clinical levels of posttraumatic stress symptoms (PTSS) or even to the development of a Posttraumatic Stress Disorder (PTSD). However, little is known about possible mid- and long-term psychological consequences in affected parents. The purpose of this study were (a) to examine the prevalence of parental birth-related PTSS and PTSD in a group of parents with VLBW preterm infants compared to parents of full-term infants 5 years after birth and (b) to investigate potential associations with risk factors for parental PTSS at 5 years postpartum. Perinatal factors (VLBW preterm or term, perceived stress during birth), psychological factors (perceived social support and PTSS 4–6 weeks postpartum, psychiatric lifetime diagnosis) and sociodemographic characteristics (number of children, singleton or multiple birth, socio-economic status), were included in the analysis. The sample consisted of 144 families (77 VLBW, 67 term birth) who participated in the prospective longitudinal cohort study “Hamburg study of VLBW and full-term infant development” (HaFEn-study) and were initially recruited at three perinatal care centers in Hamburg, Germany. PTSD prevalence and PTSS of mothers and fathers were assessed with the Impact of Event Scale-Revised (IES-R), social support with the Questionnaire of Social Support (SOZU-K-22), and lifetime psychiatric diagnoses with the Structured Clinical Interview for Diagnostic and Statistical Manual of Mental Disorders, Fourth Edition (SCID-I). Data were analyzed by hierarchic multiple regression analyses. Results showed that 5 years after birth none of the parents fulfilled the criteria for a birth-related PTSD diagnosis. For mothers, postnatal PTSS and a VLBW preterm birth significantly predicted PTSS 5 years postpartum. For fathers, psychiatric lifetime diagnosis and postnatal PTSS significantly predicted PTSS 5 years after birth. Early identification of parents with higher risk of PTSS, especially after VLBW preterm birth, and their clinical needs seems beneficial to reduce the risk of long-term consequences. More research is needed on the paternal perspective and on potential effects of preterm birth on both parents and their children's mental health outcomes.

## Introduction

The birth of a child can be perceived as traumatic, if overwhelming feelings of helplessness, fear, loss of control and pain prevail (1). A preterm birth, in particular that of a very premature (<32 gestational weeks) or very-low-birth-weight infant (VLBW, birth weight <1,500g), is regarded as an extremely stressful event and can in both parents lead to clinical levels of posttraumatic stress symptoms (PTSS) or might act as a risk factor for the development of Posttraumatic Stress Disorder (PTSD) (2, 3). The preterm birth itself usually occurs as a sudden, often unexpected event that parents face without proper mental preparation (4). However, a preterm birth is not a single traumatic incident, but can be perceived as a prolonged, more complex traumatic experience, as many challenges continue to exist for months or even years (3).

PTSS as a response to a traumatic event can manifest in 4 main clusters (a) intrusion of unwanted trauma-related memories, (b) avoidance of trauma-related thoughts or external reminders, (c) negative alterations in cognitions and mood, and (d) alterations in hyperarousal and altered reactivity. The diagnostic criteria for a PTSD are fulfilled, if symptoms from each of these clusters are reported and persist for at least 1 month (5). Elevated levels of PTSS are well-documented in the early postnatal phase after the birth of a VLBW infant [e.g., (6)]. However, little is known about possible mid- and long-term consequences. Research on this topic has focused mainly on mothers; however, fathers are affected, too, and might respond differently due to gender-related variations in birth experience and role expectations (7, 8).

In general, the prevalences of parental PTSD and PTSS vary between studies due to different diagnostic criteria of the ICD-10, DSM-IV, and DSM-5, different assessment instruments, time points and samples. For mothers of moderate to late preterm infants, Mehler et al. (9) documented a 4% PTSD rate using the Impact of Event Scale-Revised (IES-R) 3 months postpartum. Numerous other studies also have shown that mothers of preterm infants born at variable gestational ages and birth weights had higher levels of PTSS compared to mothers of term infants (6, 10–12), whereas some studies did show no differences (9).

There is some evidence on the persistence of PTSS in affected mothers. Lotterman et al. (13) reported elevated maternal PTSS from NICU hospitalization to 6 months after moderate to late preterm birth. Using the IES-R and structured clinical interviews, Kersting et al. (3) found no fully developed PTSD in mothers 6 and 14 months after birth of a VLBW infant, but PTSS significantly higher than those of mothers of term infants. On the IES-R symptom level, there was a reduction in maternal avoidance after 14 months, while intrusion persisted. Another study showed an increase in maternal PTSS from 2 weeks to 18 months postpartum (14). Gondwe et al. (15) reported no significant decrease of PTSS over 12 months in mothers of low birth weight infant and Åhlund et al. (16) showed that mothers of VLBW infants had higher PTSS 2–3 years after birth compared to mothers from the control group. This is in line with Dikmen-Yildiz et al. (2) and their finding that in women who experienced birth-related trauma, preterm birth was a risk factor for delayed onset of PTSD.

Research on fathers is scarce, however, more studies in recent years have also included their perspective. Several studies showed higher PTSS for fathers of preterm children compared to fathers of term children (6, 9, 17–19).

For fathers of moderate to late preterm children, Mehler et al. (9) reported 2% PTSD prevalence using the IES-R 3 months after birth with highest scores on the avoidance subscale. In line with these findings, Arockiasamy et al. (20) described that the primary theme for fathers having an infant on the NICU is a sense of lack of control, and some were affected to such an extent that they had to remove themselves from the situation. Lefkowitz et al. (21), using a screening questionnaire, found that 1 month after birth, 8% of fathers had PTSD with highest scores in the hyperarousal subscale; however, the sample in this study was heterogeneous since fathers whose children were on the NICU for other reasons than prematurity were included, too. Previous work that used the same data as the present study reported a prevalence of 1.4% of paternal PTSD using a structured clinical interview 4–6 weeks after birth of a VLBW infant (6).

Studies on the persistence of PTSS in fathers over time included shorter time periods compared to studies on mothers. Mehler et al. (9) described a decrease of PTSS in fathers of low-risk preterm infants from birth to 3 months postpartum. Alexander et al. (17) reported elevated PTSS in 27% of fathers of preterm infants 2–4 years after birth using the IES-R. However, since this was a small cross-sectional study, conclusions about the long-term course of PTSS cannot be drawn. To the best of our knowledge, there is no study of paternal posttraumatic stress symptomatology following the course of a VLBW preterm child's development.

According to the revised diathesis-stress model of the etiology of perinatal PTSD, there are birth-related risk factors, and also prenatal vulnerability factors and postpartum maintaining factors (22).

The birth of a VLBW infant itself can be considered as an important risk factor for parental PTSS since the confrontation with the fragile and vulnerable baby and its potentially life-threatening condition, the exhausting time of hospitalization on the neonatal intensive care unit (NICU) and worries about possible health risks and prospective disabilities in the child are challenging aspects (3, 16, 23). Furthermore, the preterm birth is often associated with both more pregnancy complications (24) and birth complications (such as operative births), that are themselves risk factors for postpartum PTSS (22).

Furthermore, perceived stress during birth is discussed as a crucial perinatal risk factor and was associated with parental PTSS 4–6 weeks postpartum (6, 25). Two meta-analyses found that a negative evaluation of the birth experience independent from objective medical risk criteria was predictive for maternal PTSD/PTSS (22, 26).

One important general risk factor is lack of social support, both during pregnancy, childbirth, and the postpartum transition into parenthood (8, 22). Perceived social support is related to PTSS (27, 28) and it might be of special importance for parents of preterm children (29). Studies found that perceived social support did not differ between mothers of preterm or term babies (9, 12, 30). Results for fathers were ambiguous; one study found no difference between fathers of preterm and term infants regarding perceived social support (9), whereas one study reported that fathers of preterm infants perceived more social support compared to fathers of term babies (30). Perceived social support was associated with PTSS shortly after birth for mothers and fathers (6), several months after birth for mothers (12), and for fathers of VLBW babies 2–4 years after birth (17).

One important prenatal vulnerability factor for postpartum PTSS is a lifetime diagnosis of a psychiatric disorder. The meta-analysis of Ayers et al. (22) demonstrated that depression in pregnancy and a history of PTSD are maternal risk factors for the development of PTSS after birth. This is in line with the meta-analysis of Grekin and O'Hara (28) who found pregnancy psychopathology and maternal psychiatric history to be associated with postpartum PTSS. Two studies demonstrated the associations between lifetime psychiatric diagnoses and PTSS ~1 month postpartum not only for mothers but also for fathers (6, 21).

One important postpartum maintaining risk factor is high postnatal PTSS levels. One study described that mothers of VLBW infants had significantly higher PTSS over the course of 14 months postpartum compared to mothers of term infants (3). Lefkowitz et al. (21) demonstrated that symptom severity of Acute Stress Disorder some days postpartum predicted PTSS 1 month postpartum in parents whose child was admitted to the NICU.

Aspects of the family and socio-economic environment might also be additional risk factors for PTSS. Evidence on parity is mixed; a meta-analysis found a significant small, positive effect of parity on postpartum maternal PTSD (22). Other studies did not report such an association for mothers (15, 31). Interestingly, having previous children was found to be associated with less PTSS in fathers of very-preterm infants ~1–2 months postpartum (31). Being parents of multiples is assumed to be more demanding and stressful than being parents of a single child (3, 32). Multifetal pregnancies carry higher risks not only of prematurity, but also of prenatal death, intrauterine growth restriction, maternal preeclampsia, diabetes, and hemorrhage during delivery (33), that all may contribute to increased stress levels.

Literature on socioeconomic status (SES) and its connection to parental PTSS is quite diverse due to different conceptualizations of the construct and local sampling particularities. Evidence on potential differences regarding factors contributing to SES between parents of VLBW infants and parents of term infants is mixed (6, 30, 34). One meta-analysis found no association between SES and maternal postpartum PTSD (22). In general, the evidence on associations between SES and PTSS or PTSD seems mixed (26).

The purpose of this study were twofold: (a) to examine the prevalence of parental birth-related PTSS and PTSD in a group of parents with VLBW preterm infants compared to parents of full-term infants 5 years after birth and (b) to investigate potential risk factors for parental PTSS 5 years postpartum: We expected the birth of a VLBW infant, higher perceived stress during birth, low perceived social support, a history of psychiatric diagnosis, and high postnatal PTSS levels to predict higher PTSS levels 5 years after birth.

## Materials and Methods

### Study Design

All data presented here stem from the prospective cohort study “Hamburg study of VLBW and full-term infant development” (HaFEn-Study), a multicenter longitudinal study from the greater Hamburg area in Germany. Families with VLBW preterm infants and families with term infants as control group were recruited between July 2006 and October 2008. The overall aim of the HaFEn-Study was to examine the association between parental mental health and child development. Questionnaires, clinical interviews, development testing, and assessment of parent-child-interaction were applied during 6 measurement points over 8 years (T1: 4–6 weeks postpartum, T2: after 6 months, T3: 12 months, T4: 24 months, T5: 4.5–5 years, and T6: after 8 years postpartum). The study followed the Declaration of Helsinki and Good Clinical Practice Guidelines, and was approved by the Ethics Committee of the Chamber of Physicians in Hamburg, Germany.

### Participants and Procedure

**Figure 1:**
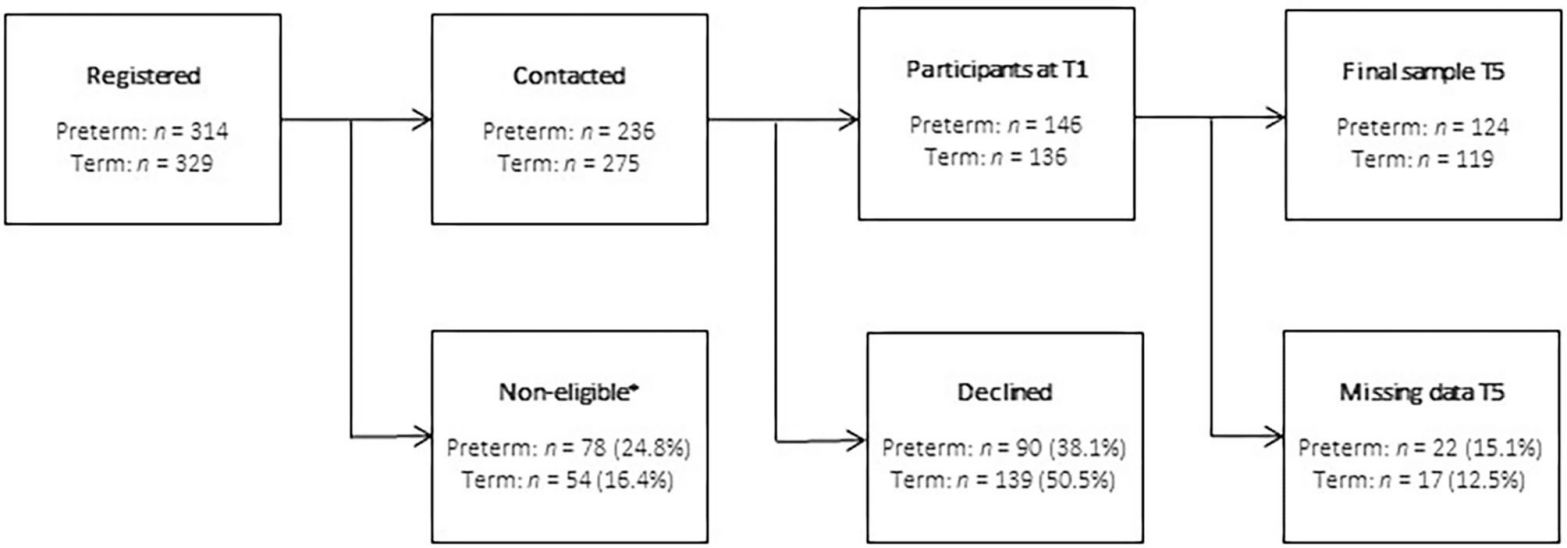
Sample selection (*non-eligible due to inability to follow study procedures, insufficient German language skills, residing too far away from study center, premature discharge, and child death before the first assessment).

The recruitment of parents with singletons and multiples took place in the three largest perinatal medical care centers in Hamburg, Germany. The following inclusion criteria were applied for the VLBW preterm group (called “preterm”): being born preterm (<37 weeks of gestation) and a birth weight of <1,500 g. Multiples were included when at least one infant was born with VLBW. All VLBW infants were born preterm. Inclusion criteria for the term group (called “term”) were ≥37 weeks of gestation for singletons and ≥34 weeks for multiples. The following exclusion criteria were applied for the preterm and term group: insufficient language skills, inability to follow study procedures, premature discharge, residing too far from the study center, and infant death before the first assessment. Four to six weeks after the birth, while the child was treated in the NICU, parents were informed about the study and asked for their participation. As a control group, parents with full-term infants born in the participating centers were selected via file withdrawal, contacted within the first week after birth and also assessed at 4–6 weeks postpartum, if interested. The sample selection is depicted in Figure 1. Informed consent was obtained. In the present study, data from T1 and T5 were analyzed. Only families in which at least one parent answered the questionnaire for the assessment of PTSS were included in the present analysis. Statistical analyses were conducted separately for mothers and fathers.

### Variables and Instruments

#### PTSD and PTSS

For the assessment of PTSS level 5 years after birth (T5) as the outcome variable and PTSS level 4–6 weeks postpartum (T1) as predictor variable, the German version of the Impact of Event Scale-Revised (IES-R) was used (35, 36). This 22 item self-report questionnaire assesses the level of posttraumatic stress symptoms based on the DSM-IV diagnostic criteria across the three PTSD categories intrusions (7 items), avoidance (8 items), and hyperarousal (7 items) during the past 7 days with higher scores representing higher levels of PTSS. The questionnaire is answered with 4 response categories (0 = “not at all” to 5 = “extremely”), with a maximum total score of 110 and higher scores indicating higher symptom levels. Additionally, a diagnostic cut-off score can be used to screen for cases with severe symptomatology. The cut-off score is based on a formula accounting the subscale scores, calculated as *X* = −0.02 × intrusion score + 0.07 × avoidance score + 0.15 × hyperarousal score – 4.36 [(35), p. 138]. A calculated score > 0.0 indicates a positive PTSD diagnosis (35). Parents in our study were asked to answer the questionnaire regarding the birth of their infant. The psychometric properties of the instrument were found to be reasonable to good (37). In the present study, the overall scale showed a good internal consistency at both assessment points for mothers (Cronbach's α = 0.88 at T1, α = 0.80 at T5) and fathers (α = 0.88 at T1 and α = 0.80 at T5).

#### Perceived Stress During Birth

Perceived stress during birth was measured with a study-specific questionnaire at T1. The questionnaire assessed how stressful the parents experienced the birth on a 5-point response scale. Maternal stress during birth was assessed with 2 items to differentiate between her perceived stress on a physical as well as on an emotional level. Paternal stress during birth was assessed by 1 item asking for the general perceived stress during birth. For comparison purposes with the paternal perspective, the mean score of both the maternal items was calculated and used for the statistical analysis. Items for mothers and fathers were answered on a scale from 0 “no stress at all” to 5 “very stressful.”

#### Perceived Social Support

The 22-item short-form of the questionnaire of social support [SOZU-K-22 (38, 39)] was used to assess perceived social support 4–6 week postpartum (T1). The items originated from 3 subscales asking for emotional support, instrumental support, social integration, and furthermore assessed satisfaction with social support and person of trust. They were being answered on a 5-point Likert scale (0 = “not true at all” to 5 = “very true”), the total sum score was used and lower scores indicate lower levels of perceived social support. The psychometric properties of the SOZU-K-22 were satisfactory (40). In the present study, internal consistency of the scale was excellent with α = 0.94 for both mothers and for fathers.

#### Psychiatric Lifetime Diagnoses

Psychiatric lifetime diagnoses (including mood, psychotic and anxiety disorders, and PTSD) were assessed with the Structured Clinical Interview for Diagnostic and Statistical Manual of Mental Disorders, Fourth Edition [SCID-I (41, 42)]. The reliability of this widely used structured clinical interview has been shown (43). For this analysis, a dichotomized variable was calculated, differentiating between participants without any symptoms (0) and those with symptoms on a clinical level in any of the above mentioned modules (1).

#### SES and Family Variables

Multiple pregnancy, the number of previous children and SES were assessed at study intake (T1) and included as control variables into the analysis. The SES was assessed with the German Winkler-Index (44) that comprises several aspects of education, occupation and income (45). The self-reported sum score ranges from 3 to 21 points with higher scores indicating a higher SES. The SES was calculated separately for mothers and fathers and was categorized into low (3–7.9), middle (8–13.8), and high (>13.9) (45).

### Statistical Analysis

First, the sample characteristics and distribution of for maternal and paternal PTSS at T5 and the control and predictor variables were investigated based on descriptive statistics. Pearson correlations were calculated to investigate the bivariate association between the variables included for the main analysis. To investigate the research question, hierarchical multiple linear regression analyses with block-wise entry were conducted. In step 1, the main model was tested with the predictor variables multiple pregnancy and number of children, birth status and perceived stress during birth, perceived social support and psychiatric lifetime diagnosis. The control variable SES was entered in step 2, and finally PTSS at T1 as a last control variable at step 3. Analyses were conducted separately for mothers and fathers. Missing values in the predictor variables were replaced using Expectation–Maximization imputation (EM algorithms). Regarding statistical power for the regression analysis, an expected minimum effect of medium size, at least *n* = 109 cases were necessary to achieve a power of 80% (Gpower 3.1.9.2). Analyses were conducted with IBM SPSS 22.

## Results

### Description of the Sample

Sociodemographic characteristics of the sample of *n* = 144 families (77 VLBW, 67 term birth), consisting of *N* = 139 mothers (*n* = 74 VLBW, *n* = 65 term birth) and *N* = 104 fathers (*n* = 50 VLBW, *n* = 54 term birth) are depicted in Table 1. Mothers had a mean age of 32.9 years (*SD* = 4.61; range = 20 to 45), whereas fathers had an average age of 35.3 years (*SD* = 5.57; range = 20 to 57). Both the maternal and paternal sample had mostly an average to high SES at T1. Five years after birth (T5), most of the participants were married to or in a relationship with the biological parent of their child. Only a small number of parents were either divorced or single parent.

**Table 1:** Sociodemographic description of the sample.

	**Mothers (*****N*** **=** **139)**		**Fathers (*****N*** **=** **104)**	
	***n***	**%**	***n***	**%**
**SES**^**a**^				
Low	8	5.8	3	2.9
Middle	56	40.3	34	32.7
High	75	54.0	67	64.4
**Marital status**^**b**^				
Single parent	7	5.0	1	1.0
Relationship with parent of child	22	15.8	17	16.3
In relationship with new partner	1	0.7	–	–
Married with parent of child	105	75.5	82	78.8
Divorced/separated	4	2.9	4	3.8
**Birth status**^**a**^				
Preterm birth	74	53.2	50	48.1
Term birth	65	46.8	54	51.9
**Parent's first born**^**a**^				
Yes	40	28.8	34	32.7
No	99	71.2	70	67.3
**Number of children**^**a**^				
One child	80	57.6	55	52.9
Two children	37	26.6	33	31.7
Three children	17	12.2	12	11.5
Four children	3	2.2	1	1.0
No information provided	2	1.4	3	2.9

a Assessed at T1;

b assessed at T5.

Among the preterm group, *n* = 57 (74.0%) families had singletons, *n* = 19 (24.7%) had twins and *n* = 1 (1.3%) had triplets. This distribution was different from the term group, in which *n* = 59 (88.1%) families had a single child and *n* = 8 (11.9%) had twins. The birth mode also differed depending on birth status. In the preterm group, the majority of children (*n* = 66; 85.7%) were delivered by a Cesarean section and *n* = 10 (13.0%) of the women gave birth vaginally. Women in the term group had fewer Cesarean sections (*n* = 20, 29.9%) and more vaginal births (*n* = 39, 58.2%; for *n* = 4 of the term group no information on birth mode was available). In the maternal sample, gestational age in the VLBW preterm group ranged from 23 to 33 weeks (*M* = 28.22, *SD* = 2.56), compared to 34 to 42 weeks (*M* = 39.08, *SD* = 1.63) in the term group. Birth weight ranged from 380 to 1,495 g (*M* = 1,061, *SD* = 297.13), and from 1,795 to 4,555 g, respectively (*M* = 3,309, *SD* = 635.89). In the paternal sample, gestational age ranged in the VLBW preterm group from 23 to 34 (*M* = 28.36, *SD* = 2.77), compared to 34 to 42 weeks (*M* = 39.09, *SD* = 1.69) in the control group. Birth weight ranged from 410 to 1,490 g, and from 1,795 to 4,300 g, respectively.

### Prevalence of PTSD and PTSS 5 Years Postpartum

In Table 2, the distribution for overall PTSS scores 5 years after birth as well as intrusion, avoidance and hyperarousal are listed. At T5, the prevalence of PTSD 5 years after birth was 0.0% in both groups of parents according to the calculated IES-R cut-off score indicating a PTSD diagnosis. Comparing the IES-R mean scale scores, mothers and fathers of preterm infants reported higher level of PTSS at T1 as well as at T5 than parents of term infants (the *SD* of the IES-R scores were higher in mothers of preterm infants compared to mothers of term infants).

**Table 2:** Descriptive statistics for the outcome variable PTSS at T5.

	**Mothers (*****N*** **=** **139)**					
	**Preterm (*****n*** **=** **74)**			**Term (*****n*** **=** **65)**		
	***M***	**(*SD*)**	**Range**	***M***	**(*SD*)**	**Range**
Overall PTSS^b^	13.84	(13.04)	0–52	6.00	(6.19)	0–20
Intrusion	5.56	(5.40)	0–21	3.26	(3.95)	0–15
Avoidance	3.35	(5.05)	0–24	0.56	(1.44)	0–7
Hyperarousal	2.95	(3.76)	0–13	0.67	(1.71)	0–9
	**Fathers (*****N*** **=** **104)**					
	**Preterm (*****n*** **=** **50)**			**Term (*****n*** **=** **54)**		
	***M***	**(*****SD*****)**	**Range**	***M***	**(*****SD*****)**	**Range**
Overall PTSS^b^	6.67	(6.56)	0–21	4.20	(5.31)	0–26
Intrusion	3.08	(3.34)	0–12	2.84	(4.18)	0–23
Avoidance	2.21	(3.59)	0–15	0.40	(1.05)	0–5
Hyperarousal	1.38	(1.74)	0–6	0.96	(2.33)	0–12

M, mean; SD, standard deviation;

b assessed at T5.

### Prediction of PTSS in Mothers and Fathers 5 Years Postpartum

Table 3 contains the descriptive statistics of the psychosocial variables included in this analysis, separately for mothers and fathers and preterm and term groups. Mothers and fathers of a preterm infant reported higher postnatal PTSS levels and higher stress during birth compared to parents of term infants. Postnatal perceived social support was comparable for mothers and fathers and slightly lower in the term compared to the preterm group. Additionally, regarding lifetime psychiatric diagnosis assessed at T1, *n* = 37 (26.6%) of mothers and *n* = 9 (8.7%) of fathers fulfilled the diagnostic criteria (for more detailed information see Supplementary Table 1).

**Table 3:** Descriptive statistics for the psychosocial predictor and control variables.

	**Mothers (*****N*** **=** **139)**					
	**Preterm (*****n*** **=** **74)**			**Term (*****n*** **=** **65)**		
	***M***	**(*SD*)**	**Range**	***M***	**(*SD*)**	**Range**
Overall PTSS^a^	23.81	(17.60)	0–69	13.30	(10.67)	0–54
Intrusion	9.30	(6.82)	0–25	7.90	(5.90)	0–22
Avoidance	7.27	(7.86)	0–28	2.16	(3.19)	0–14
Hyperarousal	7.21	(7.00)	0–28	3.21	(4.44)	0–19
Perceived stress during birth^a^	3.44	(1.65)	1–5	2.57	(1.03)	1–5
Perceived social support^a^	98.90	(11.67)	59–110	102.29	(7.56)	74–110
	**Fathers (*****N*** **=** **104)**					
	**Preterm (*****n*** **=** **50)**			**Term (*****n*** **=** **54)**		
	***M***	**(*****SD*****)**	**Range**	***M***	**(*****SD*****)**	**Range**
Overall PTSS^a^	20.36	(15.86)	0–54	14.33	(12.68)	0–57
Intrusion	8.64	(6.70)	0–26	7.98	(7.04)	0–31
Avoidance	4.56	(4.78)	0–21	1.49	(2.63)	0–13
Hyperarousal	7.15	(7.26)	0–23	4.87	(5.21)	0–20
Perceived stress during birth^a^	3.23	(1.55)	1–5	2.27	(1.27)	1–5
Perceived social support^a^	98.04	(12.11)	60–110	101.13	(7.34)	83–110

M, mean; SD, standard deviation;

a assessed at T1.

### Bivariate Correlations Between Included Variables

Zero-order correlations of the included variables are listed in Table 4. For maternal PTSS level at T5, negative small to medium-sized correlations were reported with birth status and perceived social support. Positive small to medium-sized correlations were reported for perceived stress during birth as well as with a prior psychiatric lifetime diagnosis at T1. PTSS level a T5 showed the strongest correlations with PTSS level at T1.

**Table 4:** Correlations of the included variables for mothers (above diagonal, *n* = 137) and fathers (below diagonal, *n* = 102).

		**1**	**2**	**3**	**4**	**5**	**6**	**7**	**8**	**9**
1	PTSS^b^	–	−0.12	−0.03	−0.02	−0.36^**^	0.31^**^	−0.26^**^	0.23^**^	0.57^**^
2	SES^a^	−0.04	–	0.11	0.02	0.11	−0.12	0.15^**^	−0.01	−0.16
3	Multiple pregnancy^a^ (yes/no)	0.36^**^	0.16	–	0.55^**^	−0.18^*^	−0.03	0.06	0.11	−0.06
4	Number of children^a^	0.21^*^	0.33^**^	0.48^**^	–	−0.08	−0.16	0.11	0.02	−0.11
5	Birth status^a^ (preterm/term)	−0.19	0.28^**^	−0.18	0.06	–	−0.39^**^	0.11	−0.16	−0.36^**^
6	Perceived stress during birth^a^	0.17	−0.24^*^	−0.10	−0.16	−0.37^**^	–	−0.14	0.13	0.35^**^
7	Perceived social support^a^	−0.20^*^	0.25^**^	−0.07	0.02	0.20^*^	−0.11	–	−0.12	−0.41^**^
8	Psychiatric lifetime diagnosis^a^	−0.18	−0.12	0.07	0.08	0.01	0.12	−0.01	–	0.31^**^
9	PTSS^a^	0.62^**^	−0.16	0.31^**^	0.18	−0.20^*^	0.34^**^	−0.19	0.04	–

a Assessed at T1;

b assessed at T5;

* p < 0.05,

** p < 0.01.

For fathers, small to medium-sized positive correlations were reported for PTSS level at T5 with multiple pregnancy and number of children. A small negative correlation of PTSS level at T5 was found with perceived social support. As for mothers, the strongest correlation of PTSS level at T5 was found with PTSS at T1.

Results of the multiple regression analysis are listed in Table 5. To take into account the right-skewed distribution of the outcome variable PTSS at T5 for mothers (skewness = 1.51, kurtosis = 1.92) and fathers (skewness = 1.06, kurtosis = 0.35), multiple regression analyses were conducted with bootstrapping. In total, 33% of variance in maternal PTSS at T5 was predicted by the final model. In the first step, the predictor variables explained 21% of variance in PTSS at T5 (Δ*R*^2^_*adj*_ = 0.206, *p* = 0.000), with birth status and perceived social support having significant, negative, and perceived stress during birth a significant, positive effect on PTSS at T5. At step 2, adding SES as a control variable did not significantly increase the amount of explained variance (Δ*R*^2^_*adj*_ = 0.002, *p* = 0.599). However, the effect of perceived social support was no longer significant. Adding the control variable PTSS at T1 in the third and last step explained an additional 12% of variance in maternal PTSS at T5 (Δ*R*^2^_*adj*_ = 0.122, *p* = 0.000). The effect of perceived stress during birth was no longer significant. Thus, birth status and PTSS level at T1 were the only significant predictors. Women who gave birth preterm and reported higher PTSS level at T1 also reported higher PTSS at T5.

**Table 5:** Prediction of maternal and paternal PTSS symptoms at T5.

		**Maternal PTSS at T5**					**Paternal PTSS at T5**				
**Steps**		**B**	**SE B^**+**^**	***p***	**95% CI**^**+**^		**B**	**SE B^**+**^**	***p***	**95% CI**^**+**^	
1	Constant	24.84	11.61	0.039	1.57	45.99	9.27	6.12	0.140	−2.80	21.45
	Multiple pregnancy^a^	−3.04	2.35	0.195	−7.67	1.59	**4.71**	**2.12**	**0.033**	**0.38**	**9.30**
	Number of children^a^	0.92	1.35	0.503	−1.53	3.63	0.81	1.09	0.476	−1.27	2.68
	Birth status^a^	–**5.55**	**1.76**	**0.002**	–**9.36**	–**1.95**	−0.20	1.13	0.863	−2.43	2.20
	Perceived stress during birth^a^	**1.78**	**0.72**	**0.015**	**0.35**	**3.21**	**0.80**	**0.34**	**0.024**	**0.11**	**1.48**
	Perceived social support^a^	−0.19	0.11	0.073	−0.40	0.03	−0.08	0.06	0.149	−0.19	0.03
	DSM lifetime diagnosis^a^	3.36	1.85	0.076	−0.12	6.91	–**4.26**	**1.00**	**0.001**	–**6.41**	–**2.29**
		*R^2^_*adj*_* = 0.206, *p* = 0.000					*R^2^_*adj*_* = 0.208, *p* = 0.000				
2	Constant	26.01	11.81	0.032	2.06	47.63	9.62	6.29	0.132	−2.72	21.94
	Multiple pregnancy^a^	−2.86	2.34	0.216	−7.43	1.74	**4.79**	**2.10**	**0.031**	**0.56**	**9.29**
	Number of children^a^	0.87	1.33	0.519	−1.54	3.53	0.98	1.11	0.394	−1.12	2.89
	Birth status^a^	–**5.47**	**1.77**	**0.003**	–**9.28**	–**1.88**	−0.03	1.18	0.981	−2.34	2.41
	Perceived stress during birth^a^	**1.75**	**0.73**	**0.016**	**0.28**	**3.24**	**0.78**	**0.35**	**0.027**	**0.10**	**1.46**
	Perceived social support^a^	−0.19	0.11	0.079	−0.40	0.04	−0.07	0.06	0.206	−0.18	0.04
	DSM lifetime diagnosis^a^	3.37	1.87	0.077	−0.14	6.98	–**4.44**	**1.03**	**0.001**	–**6.73**	–**2.44**
	SES	−0.12	0.19	0.537	−0.50	0.27	−0.11	0.13	0.414	−0.35	0.13
		*R^2^_*adj*_* = 0.201, *p* = 0.000					*R^2^_*adj*_* = 0.204, *p* = 0.000				
3	Constant	6.49	12.39	0.598	−17.42	28.32	5.01	5.22	0.345	−5.05	15.60
	Multiple pregnancy^a^	−1.93	2.26	0.394	−6.21	2.78	2.41	1.75	0.173	−1.06	6.11
	Number of children^a^	0.98	1.25	0.435	−1.49	3.55	0.38	0.86	0.657	−1.39	1.99
	Birth status^a^	–**3.40**	**1.52**	**0.029**	–**6.81**	–**0.14**	−0.31	1.03	0.767	−2.38	1.83
	Perceived stress during birth^a^	0.90	0.71	0.205	−0.57	2.35	0.06	0.33	0.854	−0.65	0.83
	Perceived social support^a^	−0.04	0.11	0.704	−0.27	0.20	−0.05	0.05	0.322	−0.14	0.05
	DSM lifetime diagnosis^a^	1.21	1.84	0.515	−2.37	4.98	–**3.92**	**1.04**	**0.001**	–**6.30**	–**1.93**
	SES^a^	−0.05	0.17	0.789	−0.38	0.26	0.02	0.12	0.892	−0.21	0.24
	Postnatal PTSS^a^	**0.31**	**0.08**	**0.000**	**0.15**	**0.49**	**0.22**	**0.05**	**0.000**	**0.13**	**0.31**
		*R^2^_*adj*_* = 0.325, *p* = 0.000					*R^2^_*adj*_* = 0.426, *p* = 0.000				

a assessed at T1;

b assessed at T5; bold font indicates statistical significance.

+ Standard errors and confidence intervals are based on BCa-bootstrapping with 5,000 BCa samples.

For fathers, a total of 43% of variance was predicted by the included variables. In the first step, the predictor variables explained 21% of variance in paternal PTSS at T5, with multiple pregnancy having a significant positive, and both perceived stress during birth and DSM lifetime diagnosis having a significant, negative effect on paternal PTSS at T5. Adding SES as a control variable into the model in the second step did not significantly explain additional variance in paternal PTSS at T5 (Δ*R*^2^_*adj*_ = 0.004, *p* = 0.469). Adding the control variable PTSS at T1 in the third and final step significantly explained an additional 21% of variance in PTSS at T5 (Δ*R*^*2*^_*adj*_ = 0.212, *p* = 0.000), with a positive direction of effects. In this final model, the effects of multiple pregnancy and perceived stress during birth turned insignificant. Thus, fathers with a psychiatric lifetime diagnosis and higher PTSS at T1 reported significantly higher PTSS at T5.

### Sensitivity Analyses

To investigate stability of the results, regression analyses for mothers and fathers were repeated first including only cases without missing data. Results for the significant effects were confirmed for mothers. For fathers, the effect of psychiatric lifetime diagnosis turned insignificant.

Additionally, an analysis on differences between responders and non-responders at T5 was conducted (see Supplementary Table 2) in order to examine whether T1 participants differed from T5 participants regarding age, SES, PTSS at T1, number of children, birth status, whether the child was the parent's firstborn and regarding psychiatric lifetime diagnosis. Small differences were found for maternal age, with those participating at T5 being older than those nonresponding at T5, *t*_(270)_ = −2.36, *p* < 0.05. For fathers, a small difference was found for SES, with responders having a slightly higher SES than non-responders, *t*_(223)_ = 2.34, *p* < 0.05. Significantly fewer responders fulfilled the criteria for a psychiatric lifetime diagnosis at T1 compared to non-responders, χ^2^_(2)_ = 40.24, *p* < 0.01.

## Discussion

This study aimed at examining the prevalence of parental birth-related PTSD and PTSS 5 years after the birth of a VLBW infant compared to parents of full-term infants and investigating potential risk factors for birth-related traumatic stress symptoms 5 years postpartum.

Five years postpartum, none of the parents fulfilled the cut-off criteria indicating a PTSD diagnosis based on the calculated IES-R formula (35) and the overall average level of PTSS was quite low. This finding contradicts the common view that the birth of a VLBW infant as such may often lead to ongoing parental traumatization and mental health impairment (3). Nevertheless, parents of VLBW infants were more affected by PTSS compared to parents of term infants; this is in line with other studies (3, 16). Even at 5 years postpartum, the birth status was associated with PTSS in mothers. This finding suggests that for some women, giving birth to a VLBW infant might be burdening for quite a long time.

For the mothers, one third of the variance was explained by the final model. Having a preterm VLBW infant and higher PTSS symptoms in the postnatal phase were significant risk factors for PTSS 5 years after birth for mothers. For the fathers, almost 43% of the variance was explained by the final model. Significant risk factors were a previous psychiatric lifetime diagnosis and higher PTSS symptoms shortly after birth. Interestingly, the birth status of their infant was not relevant for the fathers.

As expected, the present study showed a moderate association between birth-related PTSS at 4–6 weeks postpartum and PTSS at 5 years postpartum for mothers and fathers. Compared to the other included predictor and control variables, PTSS at 4–6 weeks postpartum explained individually the highest amount of variance for maternal and paternal PTSS at 5 years postpartum. These associations demonstrate a relevance of PTSS up to 5 years after birth. Parental posttraumatic stress can affect the development of parent-child-relationships and child outcome. For example, mothers with more PTSS were less effective in structuring interactions with their preterm children (46). Further, posttraumatic stress after preterm births in parents has been found to lead to disruptions in parenting, specifically more controlling and less sensitive parent-infant interactions, cognitive distortions, and consecutive child attachment and behavior problems (47, 48). These potential effects have to be taken into account when evaluating parental PTSS (46). Thus, high levels of parental PTSS that become evident in the NICU period may indicate a need for early clinical intervention to prevent later chronification and impaired mental health outcome on the expense of family relations and child development.

The hypothesis on the association between lifetime diagnosis of mental disorder and PTSS 5 years after birth has been confirmed in our study for fathers. Literature shows that there are barriers for men for seeking psychological support (7, 49). This knowledge and our results point to the importance of offering counseling that is suitable for these particularly vulnerable men and testing alternative ways of providing support (49).

One relatively general risk factor is the lack of social support (22). In our study, parents of a VLBW infant did not report different levels of social support compared to parents of a term infant, which is in line with previous research (9, 12, 30). Furthermore, perceived social support 4–6 weeks postpartum showed a negative bivariate association with PTSS 5 years later for both parents, but the association turned insignificant in multiple regression analysis. This result was unexpected since previous research has shown a close connection between low social support and PTSS from some weeks up to 2 years after birth (6, 12, 17). One explanation for this result might be that lack of social support played an important role for the development of PTSS after birth, as for mothers, a positive moderate sized bivariate association was found between these two variables. However, the effect on PTSS symptoms 5 years later was not strong enough when including more relevant variables into the model, like perceived stress during birth.

In contrast to recent findings and our hypothesis (6, 22), perceived stress during birth was not related to PTSS 5 years postpartum for either mothers or fathers in our study after accounting for PTSS level early after birth. Comparable to the effect for social support, this results could be due to the quite long span between birth and the assessment point. Birth-related factors seem to be associated with PTSS in the early postpartum phase (6, 50). SES, having previous children and multiple pregnancy were not associated with parental PTSS 5 years later.

Overall, average PTSS levels 5 years after birth were rather low in our sample; however, the level of PTSS varied more strongly for mothers with VLBW infants than mothers of term infants. The present study focused specifically on birth-related PTSS. It is well-documented that parents whose children were hospitalized due to preterm birth or other serious illness experienced high levels of parenting or parental stress during the time on the NICU (51–53) and some months after discharge (54, 55). This type of stress may derive from diverse sources, e.g., parental role requirements and their alterations after preterm birth, child factors like appearance, health and functional outcome, and NICU environment related stressors (56). Impairment seems to outlast for longer times compared to postpartum PTSS. Depending on the general health, mental development, and/or functional handicap of the child, long lasting consequences on parental well-being may occur (57–60). More research on the empirical as well as the theoretical level is needed for better understanding the association between general stress experienced by parents and postpartum PTSS.

### Implications

PTSS in the early postpartum phase were found to be predictive for PTSS 5 years after birth for both mothers and fathers. This certain persistence highlights the importance of early action. From a clinical perspective, screening of PTSS in both mothers and fathers of VLBW infants on the NICU is recommended (6, 17). If indicated, psychological interventions should start on the NICU in order to offer support to parents in this highly vulnerable phase that could prevent the later onset or chronic course of PTSS (61–63). A family-centered care approach and the availability of staff with psychosocial expertise in neonatology settings, that has been promoted recently across Europe and North-America, is required to serve these needs (64).

### Limitations

Our study has several limitations. First, the prevalence of PTSD was not assessed with a structured clinical interview but by applying a cut-off point on the IES-R. Second, the IES-R assesses PTSS according to the symptom clusters of the DSM-IV; thus, the fourth symptom cluster (negative alterations in cognitions and mood) introduced in the DSM-5 is not included in the present study. Third, the measurement of PTSS in postnatal women might be complicated due to some overlapping characteristics: some hyperarousal items assessed by the IES-R (e.g., sleeping or concentration problems) are commonly endorsed by postnatal women shortly after birth independent of posttraumatic stress (37). Fourth, we cannot rule out a potential selection bias due to drop-out of parents since the time of birth or to non-participation of parents at the assessment 5 years after birth. Our sensitivity analysis showed that the amount of fathers with a psychiatric lifetime diagnosis was significantly higher in the non-responder group. However, for the sociodemographic variables only few and small differences between both groups were found. Fifth, the regression analysis of parental PTSS 5 years after birth was slightly underpowered and the results should be interpreted with due caution. Sixth, we could not consider further potentially relevant variables in our analyses, like gestational age in the preterm group, or for example the health and developmental status of the 5-year-old child or whether parents had utilized professional treatment.

### Further Research Directions

Our results show that the impact of potential risk and protective factors on the course of postpartum PTSS in parents of VLBW infants should be further investigated, taking also characteristics of the child into account. Additionally, there is a need for longitudinal analyses of trajectories of postpartum PTSS in both mothers and fathers of VLBW infants and over the course of several years. This would help to gain a better understanding of the development and relevance of PTSS beyond the time after birth.

## Data Availability Statement

The datasets presented in this article are not readily available because of the ethical committee's decision. Requests to access the datasets should be directed to bindt@uke.de.

## Ethics Statement

The studies involving human participants were reviewed and approved by Ethics Committee of the Chamber of Physicians in Hamburg, Germany. The patients/participants provided their written informed consent to participate in this study.

## Author Contributions

DB managed the literature searches, interpreted the results, and drafted the manuscript. AG organized the data management, designed and conducted the statistical data analysis, and interpreted the results. CBa designed and conducted the statistical data analysis. NH participated in performing the study by both collecting data and as supervisor and interpreted the results. CBi designed the study, participated in performing the study as supervisor, managed the literature searches, and drafted the manuscript. All authors significantly contributed to and have approved the final manuscript.

## Conflict of Interest

The authors declare that the research was conducted in the absence of any commercial or financial relationships that could be construed as a potential conflict of interest.
