# Supplementary material for: Does Birth-Related Trauma Last? Prevalence and Risk Factors for Posttraumatic Stress in Mothers and Fathers of VLBW Preterm and Term Born Children 5 Years After Birth
Source: Front Psychiatry. 2020 Dec 15;11:575429. doi: 10.3389/fpsyt.2020.575429 (PMC7769835; doi:10.3389/fpsyt.2020.575429)
Supplement: Supplementary file 1 [file Data_Sheet_1.docx]

**Does birth-related trauma last?**

**Prevalence and risk factors for posttraumatic stress in mothers and fathers of VLBW preterm and term born children five years after birth**

Dana Barthel^1#^*, Ariane Göbel^1#^, Claus Barkmann^1^, Nadine Helle^1^, Carola Bindt^1^

| **Supplementary Table 1**. prevalence rates of SCID-I lifetime diagnoses at T1 | | | | | | |
| --- | --- | --- | --- | --- | --- | --- |
|  |  | Mothers (*n* = 139) | |  | Fathers (*n* = 104) | |
|  |  | *n* | % |  | *n* | % |
| Affective Disorders | | 26 | 18.7 |  | 7 | 6.7 |
| Anxiety Disorders | | 27 | 19.4 |  | 7 | 6.7 |
| PTSD | | 4 | 2.9 |  | - | - |
| Psychosis | | 1 | 0.7 |  | - | - |
| *Notes*. *N* = 3 mothers and *n* = 13 fathers did not participate in the SCID interview. For *n* = 10 mothers and *n* = 6 fathers full SCID-I information was not available. *N* = 7 mothers and *n* = 4 fathers fulfilled the diagnostic criteria for more than one diagnosis. | | | | | | |

| **Supplement Table 2**. Non-responder analysis | | | | | | | | | |
| --- | --- | --- | --- | --- | --- | --- | --- | --- | --- |
|  | Mothers | | | |  | Fathers | | | |
|  | Non-responder | | Responder | |  | Non-responder | | Responder | |
|  | *M* | (*SD*) | *M* | (*SD*) |  | *M* | (*SD*) | *M* | (*SD*) |
| Age ^I^ | 31.37 | 6.22 | 32.94 | 4.61* |  | 36.22 | 8.06 | 35.53 | 5.57 |
| SES ^I^ | 13.54 | 4.77 | 13.68 | 3.98 |  | 13.18 | 5.21 | 14.61 | 3.92* |
| Postnatal PTSS ^I^ | 21.13 | 17.49 | 18.94 | 15.73 |  | 18.23 | 17.61 | 16.84 | 14.67 |
| Number of children  ^I^ | 1.52 | 0.84 | 1.58 | 0.79 |  | 1.63 | 0.93 | 1.59 | 0.73 |
| Birth status  ^I^ | *n* | | *n* | |  | *n* | | *n* | |
| Preterm | 72 | | 74 | |  | 96 | | 50 | |
| Term | 71 | | 65 | |  | 82 | | 54 | |
| Parent’s first born  ^I^ |  |  |  |  |  |  |  |  |  |
| yes | 90 | | 99 | |  | 64 | | 70 | |
| no | 29 | | 40 | |  | 29 | | 34 | |
| DSM lifetime diagnosis ^II^ |  |  |  |  |  |  |  |  |  |
| yes | 36 | | 37 | |  | 21 | | 9* | |
| no | 86 | | 89 | |  | 62 | | 76 | |
| *Notes*. The analysis is based on data from *N* = 282 families included in the study. Analyses on differences between groups are based on independent samples t-test and χ²-test.  ^I^ questionnaire-based data from T1 (*n* = 238 mothers,  *n* = 180 fathers)  ^II^ SCID data from T1 (*n* = 269 mothers, *n* = 180); ^*^ *p* < .05. | | | | | | | | | |
